# The Ca^2+^-gated channel TMEM16A amplifies capillary pericyte contraction and reduces cerebral blood flow after ischemia

**DOI:** 10.1172/JCI154118

**Published:** 2022-05-02

**Authors:** Nils Korte, Zeki Ilkan, Claire L. Pearson, Thomas Pfeiffer, Prabhav Singhal, Jason R. Rock, Huma Sethi, Dipender Gill, David Attwell, Paolo Tammaro

**Affiliations:** 1 Department of Neuroscience, Physiology & Pharmacology, University College London, London, United Kingdom.; 2 Department of Pharmacology, University of Oxford, Oxford, United Kingdom.; 3 Center for Regenerative Medicine, Boston University School of Medicine, Boston, Massachusetts, USA.; 4 Department of Neurosurgery, National Hospital for Neurology and Neurosurgery, London, United Kingdom.; 5 Department of Epidemiology and Biostatistics, St Mary’s Hospital, Imperial College London, London, United Kingdom.

**Keywords:** Cell Biology, Vascular Biology, Calcium, Ion channels, Neurological disorders

## Abstract

Pericyte-mediated capillary constriction decreases cerebral blood flow in stroke after an occluded artery is unblocked. The determinants of pericyte tone are poorly understood. We show that a small rise in cytoplasmic Ca^2+^ concentration ([Ca^2+^]_i_) in pericytes activated chloride efflux through the Ca^2+^-gated anion channel TMEM16A, thus depolarizing the cell and opening voltage-gated calcium channels. This mechanism strongly amplified the pericyte [Ca^2+^]_i_ rise and capillary constriction evoked by contractile agonists and ischemia. In a rodent stroke model, TMEM16A inhibition slowed the ischemia-evoked pericyte [Ca^2+^]_i_ rise, capillary constriction, and pericyte death; reduced neutrophil stalling; and improved cerebrovascular reperfusion. Genetic analysis implicated altered TMEM16A expression in poor patient recovery from ischemic stroke. Thus, pericyte TMEM16A is a crucial regulator of cerebral capillary function and a potential therapeutic target for stroke and possibly other disorders of impaired microvascular flow, such as Alzheimer’s disease and vascular dementia.

## Graphical abstract

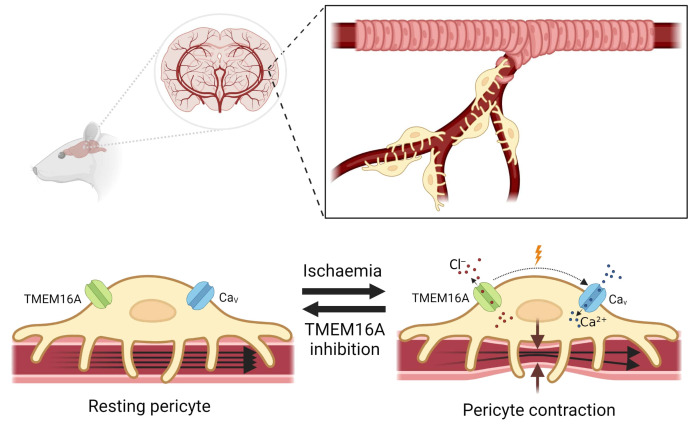

## Introduction

Cerebral blood flow (CBF) is regulated both at the arteriole and at the capillary level (1, 2). Capillaries are the site of the highest vascular resistance within the brain parenchyma, where neuronal activity can influence vessel diameter (3, 4). Cerebral capillary resistance can be altered by changes in the tone of contractile pericytes, with processes running circumferentially around the capillaries (5, 6). Electrical stimulation, optogenetically induced depolarization, contractile agonist application, and optical ablation of single cortical pericytes have demonstrated the capacity of pericytes throughout the capillary bed to contract and control capillary diameter and local blood flow (7–13).

Alterations in pericyte contraction are crucial in the pathogenesis of ischemic stroke (6), Alzheimer’s disease (11), spreading depolarization (e.g., during migraine with aura; ref. 12), and neurological problems following cardiac arrest (13). After ischemic stroke, when blood flow to the occluded artery is restored, capillaries remain underperfused (14, 15), even when thrombolysis is initiated shortly after stroke onset (16). This “no-reflow phenomenon” impairs patient recovery (17). The lack of reflow is largely due to pericyte capillary constriction during and after ischemia (6, 18), possibly as a result of the cytoplasmic Ca^2+^ concentration ([Ca^2+^]_i_) rising in pericytes after a fall of ATP level ([ATP]_i_) inhibits ion pumping; however, release of vasoconstrictors, such as endothelin-1 (ET-1) and thromboxane, in ischemic stroke may also contribute (19–21). Long-term capillary constriction will dramatically reduce local oxygen and glucose delivery, further aggravating the ischemic damage (22). For severe ischemia, pericyte-evoked capillary constriction is followed by pericytes dying in rigor (6, 18), thus prolonging the decrease of CBF. This pericyte loss (23), which is partly caused by the [Ca^2+^]_i_ rise that triggers contraction (6, 18), damages the blood-brain barrier (BBB) (24–27). Neutrophil stalling in capillaries, which may be a result of pericyte-mediated narrowing of the capillary lumen, has also been proposed as a factor contributing to the no-reflow phenomenon after stroke (16, 28).

Preventing pericyte contraction could be of clinical benefit, but the determinants of pericyte tone are poorly understood. Pericyte contractility is controlled by [Ca^2+^]_i_ (29–31), which can be raised by depolarization-activated Ca^2+^ (Cav) channels or by G_q_ protein–coupled receptors (G_q_PCRs) triggering Ca^2+^ release from intracellular stores. However, Ca^2+^-activated Cl^–^ channels (specifically, TMEM16A; refs. 32–34) are found in smooth muscle and pericytes (35–38) and may be activated by any [Ca^2+^]_i_ rise that triggers contraction. Because smooth muscle cells (SMCs) and pericytes have a high intracellular Cl^–^ concentration (set by the plasma membrane Na^+^-K^+^-2Cl^–^ cotransporter NKCC1 and the Cl^–^/HCO_3_^–^ exchanger AE2; refs. 35–37), when Cl^–^ channels open, the resulting Cl^–^ efflux will cause a depolarization (for example, in kidney pericytes, ref. 37, the Nernst potential for Cl^–^ is around –30 mV). This depolarization is expected to activate Cav channels and amplify the increase in [Ca^2+^]_i_ and contraction that occur (39). In this paper, we test the hypotheses (a) that TMEM16A is a depolarizing force in cerebral pericytes during agonist stimulation and ischemia and (b) that inhibition of this channel opposes capillary constriction, thus reducing tissue damage during ischemia.

Using experiments in brain slices and in vivo, we demonstrate here that TMEM16A is a crucial amplifier of cortical pericyte contraction evoked by [Ca^2+^]_i_ rises triggered by physiological modulators and ischemia. The importance of this is emphasized by genetic analysis that implicated TMEM16A expression level as a determinant of patient recovery after stroke. Pharmacological inhibition of TMEM16A reduced the ischemia-evoked contraction and death of pericytes, improved postischemic CBF, decreased capillary neutrophil blocks at pericyte somata, and reduced brain hypoxia and infarct size after ischemia, thus highlighting TMEM16A inhibition as a therapeutic strategy to improve reflow after stroke and other conditions of impaired microvascular blood flow.

## Results

### TMEM16A generates Ca^2+^-activated Cl^–^ currents in cortical pericytes.

**Figure 1:**
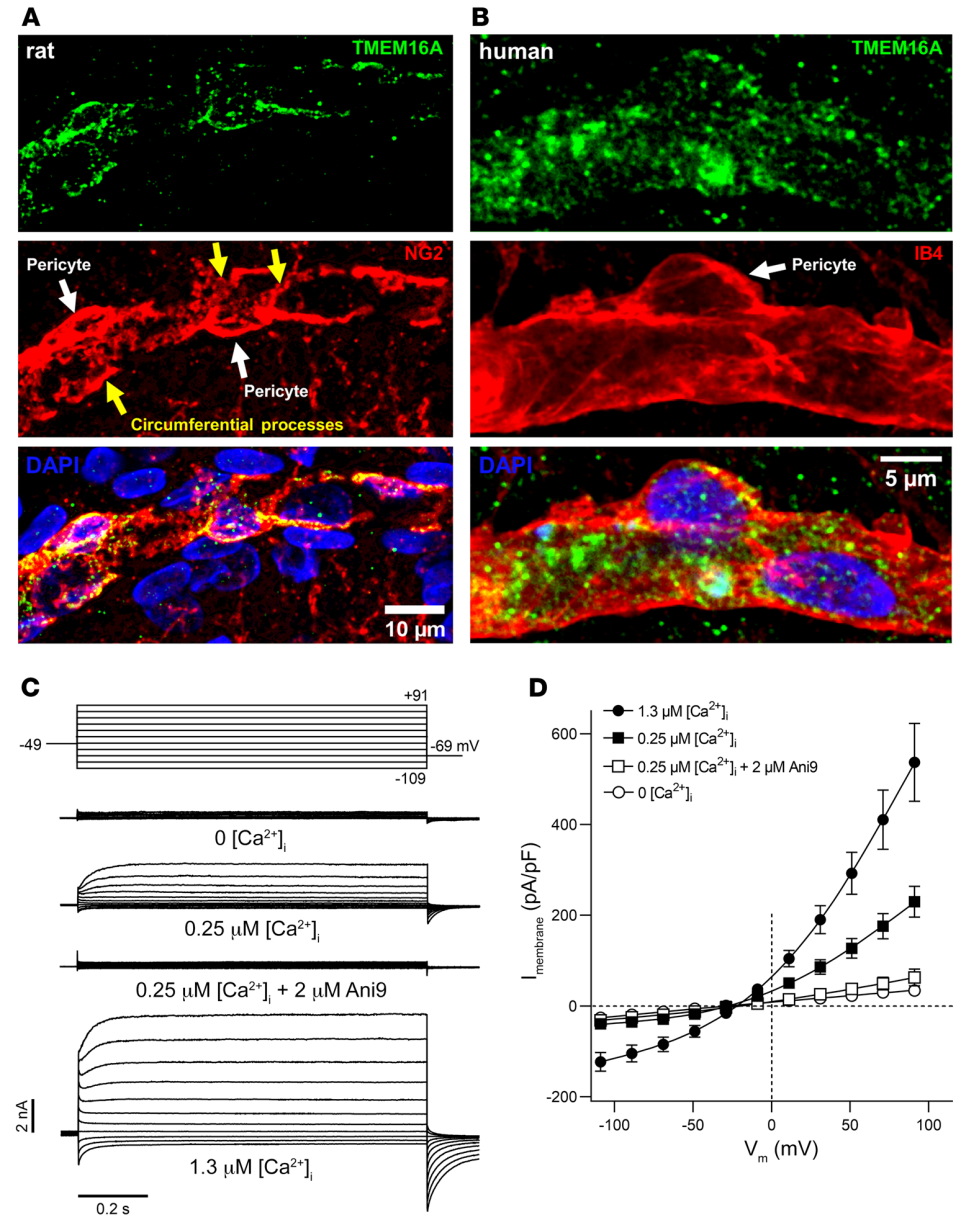
Cortical pericytes express functional TMEM16A channels. (**A**) TMEM16A expression in the soma and circumferential processes (yellow arrows) of NG2-labeled pericytes (white arrows) in a representative fixed cortical slice of a P21 rat. Scale bar: 10 μm. (**B**) TMEM16A expression in a pericyte labeled using isolectin B4 (IB4) in a fixed cortical slice of a 40-year-old human (representative of data from 5 participants). Scale bar: 5 μm. (**C**) Representative family of whole-cell TMEM16A currents recorded from individual rat cortical pericytes, using pipette solutions designed to isolate Cl^–^ currents and various free [Ca^2+^]_i_ (nominally 0, 0.25, or 1.3 μM), in the absence or presence of Ani9 (2 μM). The voltage protocol is illustrated at the top, shown after correction for liquid junction potential. (**D**) Mean whole-cell TMEM16A current density versus voltage relationships in cortical pericytes (*n =* 9–14), with various [Ca^2+^]_i_, in the absence or presence of Ani9.

TMEM16A mRNA is strongly expressed in cerebral pericytes and SMCs (38, 40), but the presence of TMEM16A protein in cerebral pericytes has not been previously assessed. We examined TMEM16A expression in pericytes of rat cortical slices and in human cortical tissue removed surgically to access underlying tumors. Pericytes were labeled with antibodies against the proteoglycan NG2 (which is expressed by pericytes; ref. 41) and TMEM16A, and the basement membrane around pericytes was visualized using isolectin B4 conjugated to an Alexa dye. Consistent with that in transcriptome studies (Supplemental Figure 1A; supplemental material available online with this article; https://doi.org/10.1172/JCI154118DS1), 90% of the TMEM16A expression was in pericytes (Figure 1, A and B, and Supplemental Figure 1B). SMCs also expressed TMEM16A (Supplemental Figure 1C).

To assess the importance of TMEM16A, pericytes were whole-cell clamped in rat cortical slices, with a Cs-based internal solution that enables recording of Cl^–^ currents (see Methods). With an intracellular solution containing 0.25 μM free [Ca^2+^]_i_, the steady-state current-voltage relationship was outwardly rectifying (Figure 1, C and D), with a reversal potential (–27.2 ± 4.2 mV, *n =* 14) near the value of E_Cl_ (–25 mV). When [Ca^2+^]_i_ was raised to 1.3 μM, the membrane current on either side of E_Cl_ was increased, and its rectification was reduced, as observed for cloned TMEM16A channels (42, 43). With 0.25 μM [Ca^2+^]_i_, the TMEM16A inhibitor Ani9 [2-(4-Chloro-2-methylphenoxy)-*N*′-(2-methoxybenzylidene)acetohydrazide] reduced the current (by 73% at 100 mV, *P =* 0.02, Kruskal-Wallis test with Dunn’s multiple comparisons test) to a level only slightly larger than when using Ca^2+^-free intracellular solution, for which TMEM16A channels should be closed (Figure 1D). Near the pericyte resting potential (~–40 mV), with 0.25 or 1.3 μM [Ca^2+^]_i_, Ca^2+^-activated Cl^–^ channels contributed a conductance of approximately 5 or 21 nS, respectively, to the cell. This is larger than the cell conductance, with a normal K^+^-based internal solution and nominally 0 [Ca^2+^]_i_ (~0.9 nS, see below), implying that TMEM16A has considerable scope to alter the membrane potential of the cell.

### Pericyte contraction evoked by G_q_PCR activation requires Ca^2+^ entry via Cav channels.

**Figure 2:**
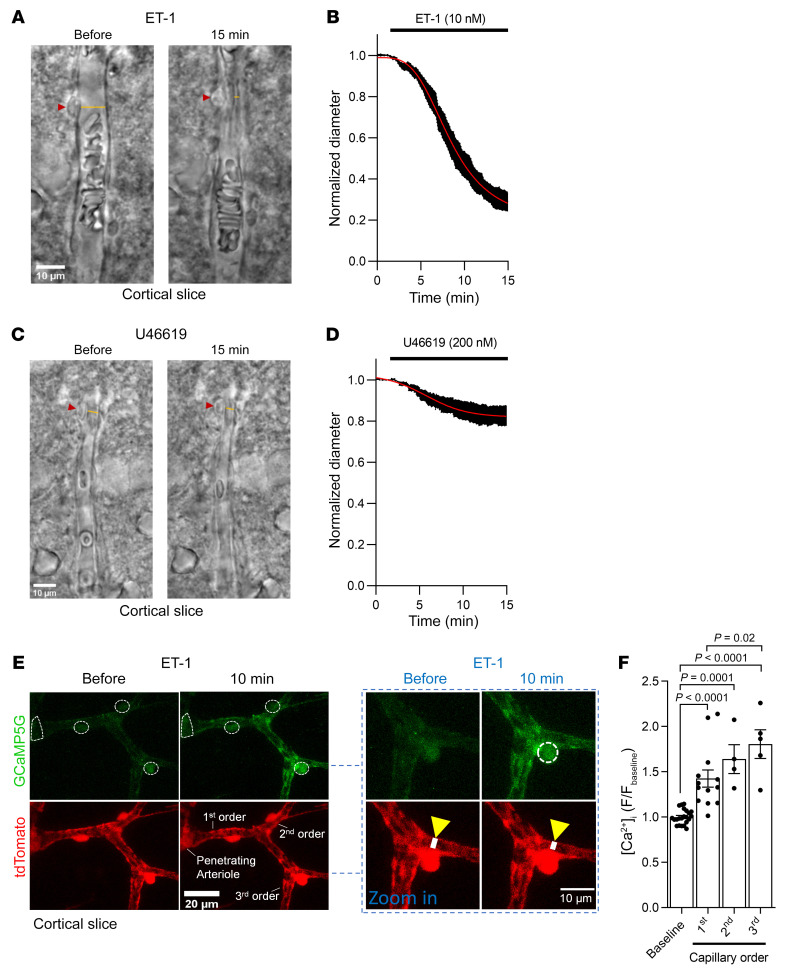
Vasoconstricting G_q_PCR agonists raise pericyte [Ca^2+^]_i_ and constrict capillaries at pericyte somata in acute cortical slices. (**A**) Representative bright-field images of a live rat cortical capillary pericyte before and after 15 minutes exposure to endothelin 1 (ET-1; 10 nM). Red arrowheads indicate the pericyte soma, and yellow lines indicate where the internal capillary diameter was measured. Scale bar: 10 μm. (**B**) Mean internal capillary diameter at pericyte somata during exposure to ET-1 (10 nM), normalized to the diameter measured in the absence of ET-1 (*n =* 10). The ET-1–evoked capillary constriction was not dependent on the sex of rats (Supplemental Figure 3A). (**C**) Representative bright-field images of a live capillary pericyte, as in **A**. The thromboxane A2 analog U46619 (200 nM) was applied. Scale bar: 10 μm. (**D**) Mean internal capillary diameter at pericyte somata during exposure to U46619 (200 nM), normalized to the diameter measured in the absence of U46619 (*n =* 8). (**E**) Two-photon microscopy images (maximum intensity projections) of SMCs on a PA and pericytes on first- to third-order capillary branches in acute cortical slices obtained from NG2-Cre^ERT2^-GCaMP5G mice. ET-1 raised the somatic [Ca^2+^]_i_ of SMCs and pericytes (encircled with white dashed lines). Scale bar: 20 μm. The pericyte [Ca^2+^]_i_ rise coincides with capillary constriction, as indicated by the white line across the vessel lumen in the higher magnification image (scale bar: 10 μm). (**F**) ET-1 significantly raises [Ca^2+^]_i_ in first- to third-order pericyte somata and evoked the greatest [Ca^2+^]_i_ rise in third-order pericytes. The mean GCaMP5G fluorescence in pericyte somata (points indicate individual pericytes from 5 mice; baseline, *n =* 22; first, *n =* 13; second, *n =* 4; third, *n =* 5) was normalized to the mean GCaMP5G fluorescence of the 17 minutes baseline (F_baseline_) with aCSF (1-way ANOVA with Tukey’s post hoc test).

Pericytes in acute rat cortical slices constricted capillaries in response to the contractile agonists ET-1 and U46619 (a thromboxane A_2_ analog), which act on G_q_PCRs (9, 11). The circulating levels of ET-1 and thromboxane A_2_ are increased during stroke (19–21, 44). The capillary diameter at pericyte somata was reduced by approximately 70% (*P <* 0.0001, paired 2-tailed Student’s *t* test) and approximately 20% (*P <* 0.0001, paired 2-tailed Student’s *t* test) by ET-1 (10 nM) and U46619 (200 nM), respectively (Figure 2, A–D).

The [Ca^2+^]_i_ rise evoked by ET-1 in pericytes was assessed using 2-photon imaging of mice expressing tdTomato and the Ca^2+^ indicator GCaMP5G, driven by the promoter for NG2 (NG2-Cre^ERT2^-GCaMP5G mice, see Methods). ET-1 increased [Ca^2+^]_i_ in pericytes on (at least) the first-, second-, and third-order branch capillaries from penetrating arterioles (PAs), where first order refers to the first branch off the PA, and second order refers to a branch off the first order, etc. (Figure 2, E and F). The [Ca^2+^]_i_ response to ET-1 increased significantly from the first- to the third-order branch pericytes (Figure 2F). We show below that these differences in [Ca^2+^]_i_ rise are not due to a different ET-1 receptor response per se but may reflect different numbers of TMEM16A and/or Cav channels in pericytes of different branch orders.

**Figure 3:**
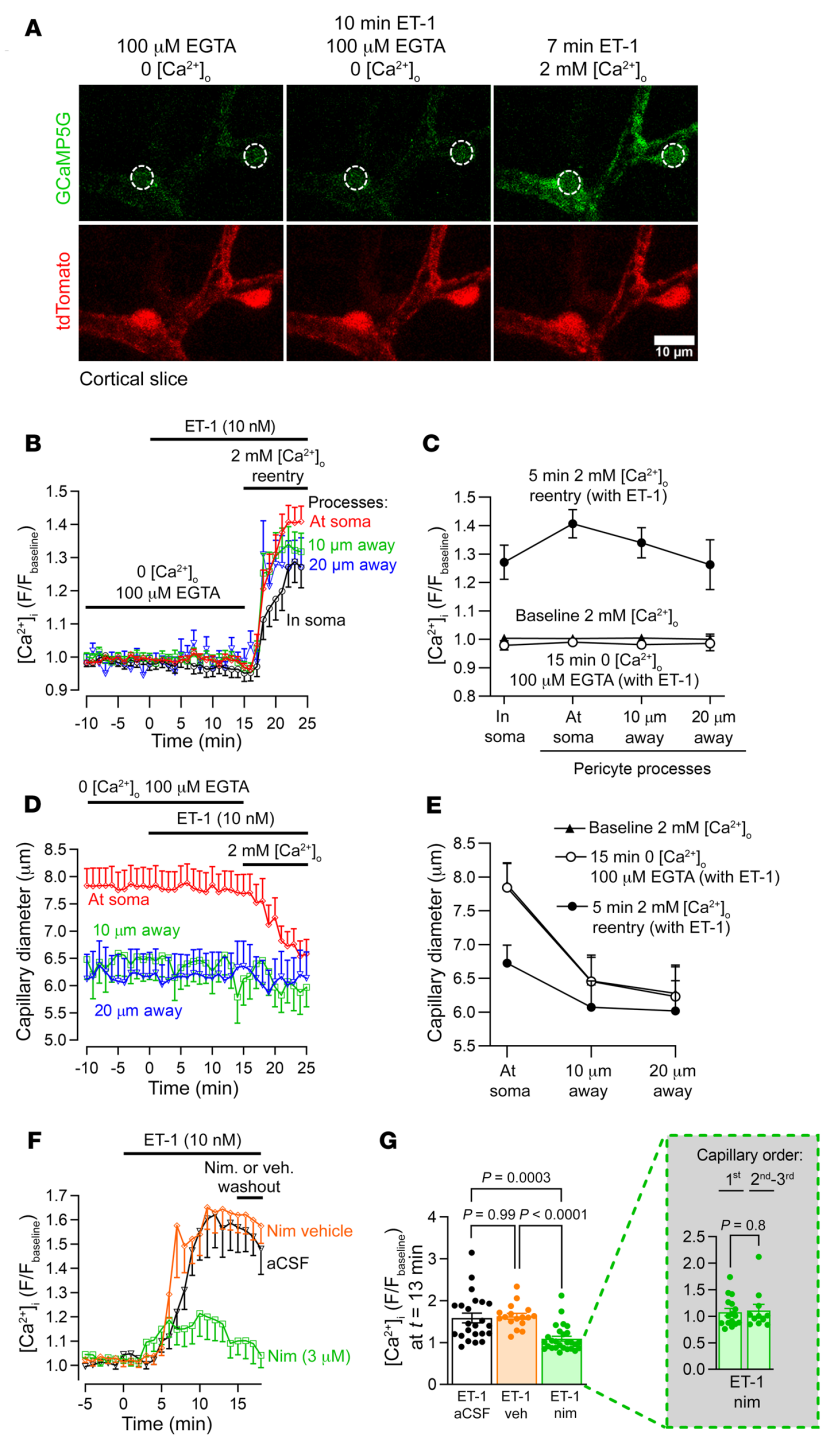
Pericyte contraction evoked by G_q_PCR activation requires Ca^2+^ entry via Cav channels. (**A**) Two-photon microscopy images (maximum intensity projections) of pericytes on first- to third-order capillary branches from the PA in an acute cortical slice of a NG2-Cre^ERT2^-GCaMP5G mouse. Dashed white circles denote pericyte somata. Scale bar: 10 μm. (**B**) Removing extracellular Ca^2+^ abolished the ET-1–evoked [Ca^2+^]_i_ rise. Time course of GCaMP5G fluorescence (F) in pericyte somata (red trace; *n =* 32) and processes (other traces; *n =* 79) normalized to mean baseline fluorescence with 2 mM [Ca^2+^]_o_ (the last 10 minutes of 15 minutes in 0 [Ca^2+^]_o_ are shown). [Ca^2+^]_i_ changes in processes were quantified less than 5 μm from pericyte somata centers (“at soma”) and at 10 μm and 20 μm along the vessel from the soma center. (**C**) 15 minutes of 0 [Ca^2+^]_o_ did not affect pericyte [Ca^2+^]_i_ (compare bottom 2 plots). Reintroducing Ca^2+^ in the continuous presence of ET-1 raised pericyte [Ca^2+^]_i_ in processes and somata (see also, **B**). (**D**) Capillary constriction at pericyte somata (*n =* 40) coincides with the [Ca^2+^]_i_ rise upon 2 mM [Ca^2+^]_o_ reperfusion in **B**. There was no significant change in capillary diameter away from pericyte somata at 10 μm (*n =* 18) or 20 μm (*n =* 11). (**E**) Capillary diameter is larger at baseline and constricts in response to 2 mM [Ca^2+^]_o_ reperfusion at pericyte somata. In **D** and **E**, diameter is from tdTomato channel. (**F**) Time course of ET-1–evoked [Ca^2+^]_i_ change in pericyte somata (*n =* 23), normalized to aCSF baseline. Nimodipine (3 μM) (*n =* 27) or vehicle (*n =* 17) were applied 15 minutes before ET-1 application. (**G**) Nimodipine greatly attenuated the ET-1–evoked pericyte [Ca^2+^]_i_ rise (note that 0 [Ca^2+^]_i_ is at 1 on the *y* axis) (Kruskal-Wallis test with Dunn’s post hoc test). The inset shows that in the presence of nimodipine, the ET-1–evoked [Ca^2+^]_i_ rise was similar in pericytes on first-order (*n =* 9) versus second- and third-order branches (*n =* 10) (unpaired 2-tailed Student’s *t* test).

Ca^2+^ released from stores by G_q_PCR stimulation may activate actomyosin directly or may indirectly (via activation of TMEM16A) evoke depolarization and, thus, activate Cav channels, causing an influx of extracellular Ca^2+^ ([Ca^2+^]_o_) and myofilament contraction. To distinguish between these possibilities, we applied ET-1 in the absence of [Ca^2+^]_o_. At 0 [Ca^2+^]_o_, ET-1 neither significantly raised [Ca^2+^]_i_ (Figure 3, A–C), nor evoked pericyte contraction (Figure 3, D and E). Reintroducing [Ca^2+^]_o_ with ET-1 present rapidly raised [Ca^2+^]_i_ (Figure 3, B and C) in the pericyte soma (*P =* 6.2 × 10^–6^, paired 2-tailed Wilcoxon’s test with continuity correction) and processes (*P <* 2.2 × 10^–16^, paired 2-tailed Wilcoxon’s test with continuity correction), presumably via [Ca^2+^]_o_ entry (see below). This [Ca^2+^]_i_ rise evoked a significant capillary constriction at the soma of the pericytes (*P =* 0.04, paired 2-tailed Wilcoxon’s test with continuity correction) (Figure 3, D and E), where most circumferential processes are located (10), but not at 10 μm (*P =* 0.07, paired 2-tailed Student’s t test) or 20 μm (*P =* 0.5, paired 2-tailed Student’s t test) along the capillary from the pericyte soma (Figure 3E), where the processes run more longitudinally (11). Capillary diameter was larger at the soma than away from the soma (Figure 3E) in the absence of ET-1 (*P =* 0.002, unpaired 2-tailed Student’s t test) and also after 15 minutes in 0 [Ca^2+^]_o_ and ET-1 (*P =* 0.003, unpaired 2-tailed Student’s t test), consistent with measurements in vivo (6), suggesting that the pericyte soma may release factors that induce growth of the endothelial tube.

To test whether the absence of an ET-1–evoked pericyte [Ca^2+^]_i_ rise and contraction in 0 [Ca^2+^]_o_ was the result of preventing Ca^2+^ entry via Cav channels, or alternatively a result of depleting internal stores of Ca^2+^, we examined the effect of the L-type Cav blocker nimodipine on the ET-1 response in normal [Ca^2+^]_o_ solution. Nimodipine (3 μM) inhibited [Ca^2+^]_i_ rise by 86% (Figure 3, F and G), implying that most Ca^2+^ enters via Cav channels (ET-1 can also activate, for example, TRPM4 and TRPC3, TRPC5, TRPC6, and TRPC7 channels in other cell types; however, the 86% suppression of the steady-state [Ca^2+^]_i_ rise by nimodipine in Figure 3G implies that Cav channels contribute the great majority of the Ca^2+^ influx.). The [Ca^2+^]_i_ rise seen in nimodipine was similar in first-, second-, and third-order pericytes (Figure 3G), suggesting that heterogeneity in the [Ca^2+^]_i_ rise between first- and third-order pericytes (Figure 2F) may involve differences in the number of L-type Cav channels activated downstream of the small [Ca^2+^]_i_ rise caused by ET-1 receptors alone.

Together these data demonstrate that most of the ET-1–triggered contraction requires Ca^2+^ entry via Cav channels. This raises the question of how Ca^2+^ released from internal stores by ET-1 leads to Cav activation.

### TMEM16A amplifies the pericyte [Ca^2+^]_i_ rise and contraction evoked by vasoconstrictors.

**Figure 4:**
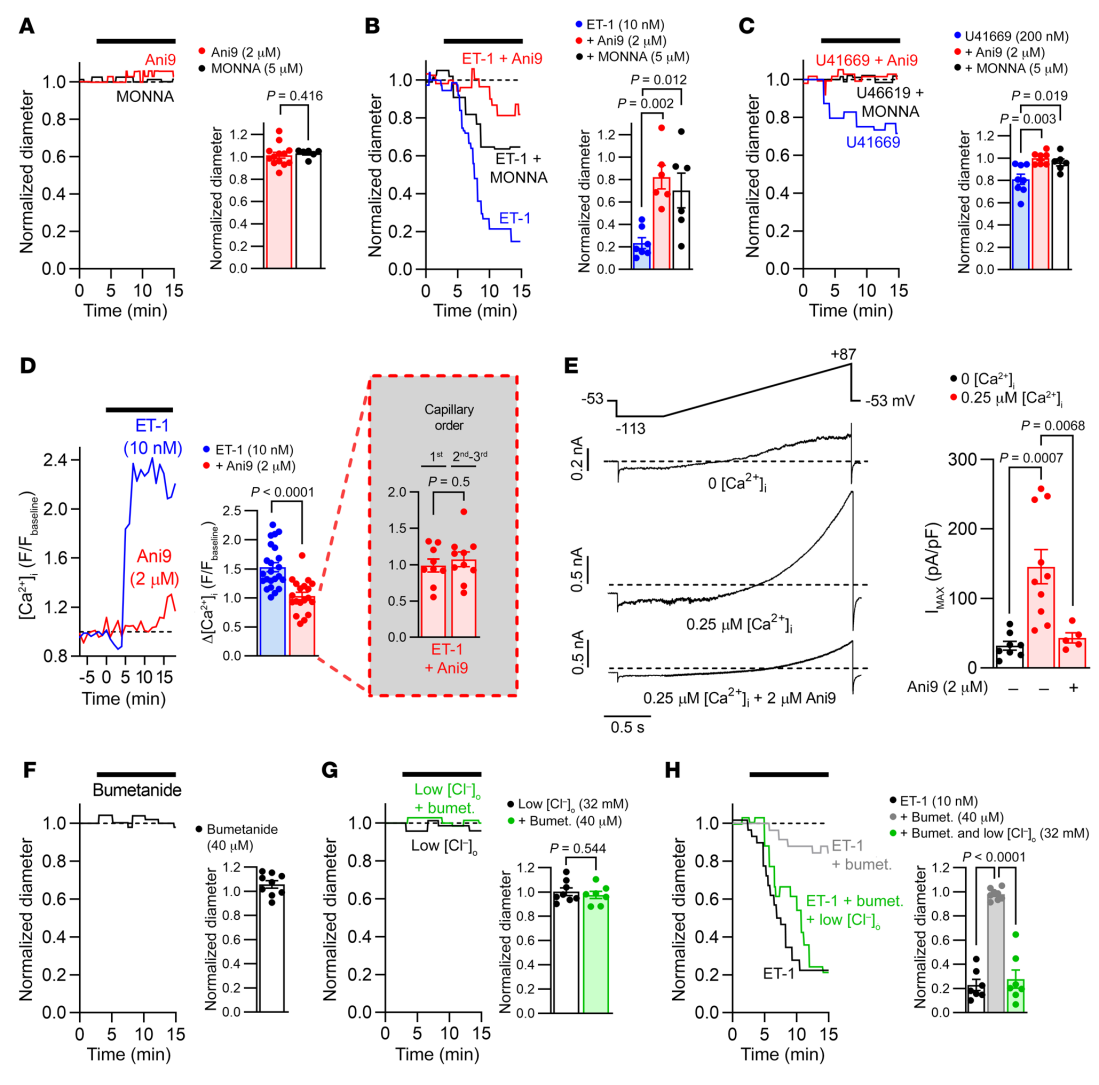
Effects of Cl^–^ on TMEM16A-mediated control of pericyte [Ca^2+^]_i_ and tone. (**A**) Capillary diameter at pericyte somata during Ani9 (2 μM) or MONNA (5 μM) superfusion in acute rat cortical slices, normalized to baseline diameter (left). Mean normalized capillary diameter after exposure to Ani9 (*n =* 13) or MONNA (*n =* 6) (right). (**B** and **C**) Ani9 or MONNA reduced capillary constriction evoked by (**B**) ET-1 (10 nM) or (**C**) U46619 (200 nM) (*n =* 6–8). (**D**) Ani9 (*n =* 19) reduced the ET-1–evoked [Ca^2+^]_i_ rise in pericyte somata (*n =* 23) in cortical slices of NG2-Cre^ERT2^-GCaMP5G mice. GCaMP5G fluorescence (F) was normalized to baseline fluorescence in aCSF. The inset shows that in the presence of Ani9, the ET-1–evoked [Ca^2+^]_i_ rise was similar in pericytes on first-order (*n =* 9) versus second- and third-order (*n =* 10) branches from the PA. (**E**) Whole-cell currents of rat cortical pericytes in acute rat cortical slices (left). The voltage protocol is illustrated at the top, shown after correction for the liquid junction potential. Free [Ca^2+^]_i_ was 0 or 0.25 μM. Mean whole-cell current density (I_MAX_) at the end of the ramp (i.e., +87 mV) in 0 (*n =* 8) or 0.25 μM (–Ani9: *n =* 10; +Ani9: *n =* 5) [Ca^2+^]_i_ (right). (**F**) Normalized capillary diameter at pericyte somata during bumetanide (40 μM) superfusion (left). Mean normalized capillary diameter at pericyte somata after exposure to bumetanide (40 μM) (*n =* 9) (right). (**G**) Effects of low [Cl^–^]_o,_ (*n =* 7) and bumetanide (*n =* 8) on rat cortical capillary diameter. (**H**) Changes in capillary diameter in response to ET-1 after 15 minutes of preincubation with bumetanide (*n =* 7) with (*n =* 7) or without (*n =* 8) a lower [Cl^–^]_o_. (**G** and **H**) Representative capillary responses (left). Normalized capillary diameter (right). Numbers of animals are detailed in Supplemental Table 2. (**A**) Mann-Whitney test; (**D** and **G**) unpaired 2-tailed Student’s *t* test; (**B**, **C**, **E**, and **H**) 1-way ANOVA with Bonferroni’s post hoc multiple comparisons test.

Two structurally unrelated TMEM16A inhibitors, 2-[(4-Methoxy-2-naphthalenyl)amino]-5-nitro-benzoic acid (MONNA) (45) and Ani9 (46), did not affect the diameter of cortical capillaries when they were not exposed to exogenous constrictors (Figure 4A), consistent with the idea that TMEM16A channels have no or low activity in the absence of substantial G_q_PCR stimulation. Lack of basal TMEM16A activity may reflect a decrease in vascular tone in brain slices, possibly as a result of a lack of blood flow and the shear stress it generates, or lack of noradrenaline release from the axons of locus coeruleus neurons (which become severed in the brain slicing procedure). However, MONNA and Ani9 greatly reduced the capillary constriction evoked by ET-1 (10 nM) (Figure 4B) and U46619 (200 nM) (Figure 4C). Furthermore, Ani9 strongly attenuated the ET-1–evoked [Ca^2+^]_i_ rise in all tested capillary branch orders (Figure 4D). The 94% reduction of the ET-1–evoked [Ca^2+^]_i_ rise produced by Ani9 shown in Figure 4D, where fluorescence change was measured from the *F/F_baseline_* value of 1, is not inconsistent with the approximately 75% reduction of TMEM16A current shown in Figure 1D: there is no reason to expect a linear relationship between the suppression of the [Ca^2+^]_i_ rise and the suppression of the TMEM16A current, because of the nonlinear relationships that exist between [Ca^2+^]_i_ and TMEM16A activation and between TMEM16A-evoked depolarization and voltage-gated Ca^2+^ current activation. These results are consistent with a TMEM16A-mediated depolarization, amplifying the ET-1–evoked rise of [Ca^2+^]_i_ by activating Cav channels.

The Ani9-mediated reduction of the ET-1–evoked [Ca^2+^]_i_ rise and pericyte contraction is unlikely to result from Ani9 indirectly affecting pericyte tone by acting on nearby endothelial cells, astrocytes, or neurons, because TMEM16A is nearly exclusively expressed in cortical pericytes and SMCs (Figure 1, A and B; Supplemental Figure 1, A–C; and refs. 47, 48). Furthermore, in whole-cell clamped pyramidal neurons, the action potential response to current injection, the resting potential, and the neuronal input resistance were not affected by Ani9 (Supplemental Figure 2).

We confirmed that TMEM16A was present at sufficient density to significantly change the membrane potential of the cell and, thus, activate Cav channels, by whole-cell clamping pericytes with physiological internal solutions containing 0 or 0.25 μM free [Ca^2+^]_i_ (see Methods). With 0 free [Ca^2+^]_i_, the resting potential and input resistance were –42.0 ± 4.5 mV and 1.1 ± 0.5 GΩ, respectively, in 8 cells. With 0.25 μM [Ca^2+^]_i_, an inward current of –12.8 ± 2.3 pA/pF (*n =* 10) (mean cell capacitance, 10.6 ± 1.0 pF [*n =* 10]) was present at –40 mV, increased from a value of +0.3 ± 1.2 pA/pF (*n =* 8) in 0 [Ca^2+^]_i_ (mean cell capacitance: 10.3 ± 1.0 pF [*n =* 8]). In the presence of 2 μM Ani9, this current was reduced to –5.3 ± 0.7 pA/pF (*n =* 5) (mean cell capacitance, 11.4 ± 1.6 pF [*n =* 5]), implying a TMEM16A-mediated Cl^–^ conductance of approximately 5 nS. This Ca^2+^-activated current depolarized the resting potential to –18.3 ± 4.1 mV (*n =* 10) (Figure 4E).

### Pericyte tone is strongly influenced by the transmembrane Cl^–^ gradient.

If TMEM16A confers a fundamental depolarizing mechanism recruited during G_q_PCR activation, altering the transmembrane Cl^–^ gradient should alter the amplification of constriction produced by TMEM16A. This gradient was altered in cortical slices using 3 different strategies: (a) exposing slices to bumetanide (40 μM) to inhibit the Cl^‑^ importer NKCC1 and, thus, reduce the intracellular Cl^–^ concentration ([Cl^–^]_i_) and the depolarizing influence of TMEM16A; (b) reducing the extracellular Cl^–^ concentration ([Cl^–^]_o_) to increase TMEM16A-mediated depolarization; or (c) a combination of these treatments. In slices not exposed to a G_q_PCR agonist, alterations in the Cl^–^ gradient did not affect capillary diameter (Figure 4, F and G), consistent with TMEM16A channels being closed under these conditions. In contrast, when pericytes were exposed to ET-1 following pretreatment with bumetanide (to reduce the depolarizing [Cl^–^]_i_ gradient), the resulting capillary constriction was attenuated approximately 4-fold (Figure 4H). Lowering [Cl^–^]_o_ to reinstate a depolarizing Cl^–^ gradient in the presence of bumetanide restored the ET-1–evoked capillary pericyte contraction (Figure 4H) to a magnitude similar to that seen with ET-1 application alone (Figure 4B). Thus, capillary pericyte contraction is strongly dependent on the transmembrane Cl^–^ gradient.

### TMEM16A KO and raising [K^+^]_o_ confirm that TMEM16A amplifies constrictions.

**Figure 5:**
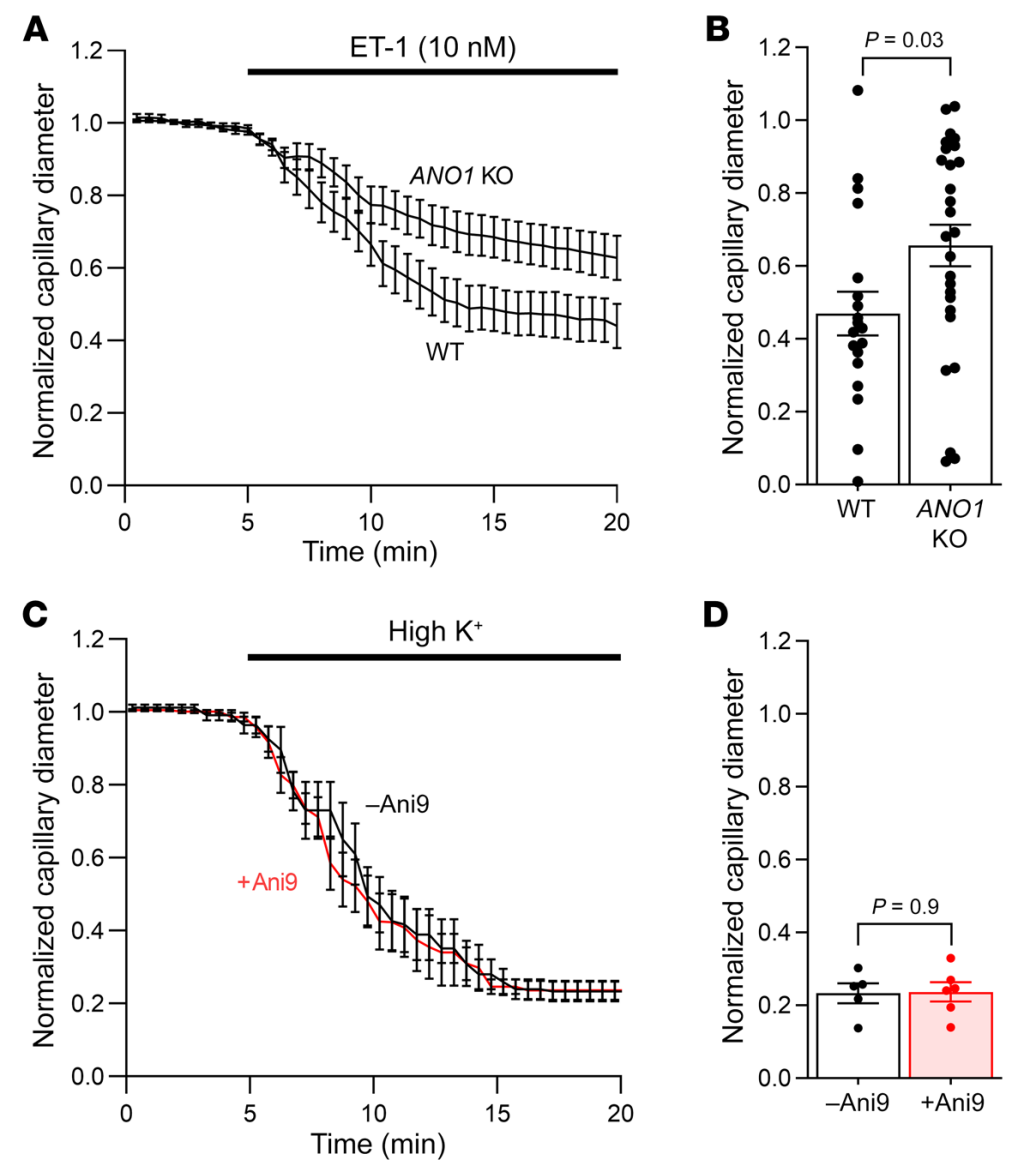
TMEM16A KO in pericytes reduces endothelin-1–evoked pericyte contraction, and depolarizing the membrane potential induces pericyte contraction independent of TMEM16A block. (**A**) Mean internal capillary diameter at pericyte somata during exposure to endothelin-1 (ET-1) (10 nM), normalized to the diameter measured in the absence of ET-1, in acute cortical slices of wild-type or *ANO1*-KO mice. (**B**) TMEM16A KO reduced the average ET-1–evoked pericyte contraction during the last 5 minutes of the experiment (Ano KO, *n =* 27; Wild-type, *n =* 19). (**C**) Mean internal capillary diameter at pericyte somata during exposure to 92.5 mM extracellular potassium ([K^+^]_o_), normalized to the diameter measured in the presence of 2.5 mM [K^+^]_o_ in acute rat cortical slices. (**D**) Raising [K^+^]_o_ evokes pericyte contraction, and this is not reduced by Ani9 (2 μM). Points indicate individual pericytes from 5 rats per condition (–Ani9: *n =* 5; +Ani9: *n =* 6). (**B** and **D**) Unpaired 2-tailed Student’s *t* test.

Using wild-type mice or floxed TMEM16A-KO mice (TMEM16A is also known as *ANO1*) crossed with NG2-Cre^ERT2^ mice (to KO TMEM16A in NG2-expressing pericytes), we assessed the effect of ET-1 on the pericyte-mediated capillary constriction. This showed that KO of TMEM16A reduced the ET-1–evoked constriction of capillaries by pericytes (Figure 5, A and B), as was also seen when using pharmacological inhibition of TMEM16A. The smaller effect of the KO in Figure 5A, compared with pharmacological inhibition in Figure 4B, likely reflects the incomplete KO achieved with Cre (the reported percentage of cells, probably oligodendrocyte precursor cells, that undergo recombination was approximately 60% at 2 weeks after tamoxifen administration for the Cre line we used, ref. 49, although in retinal pericytes using a different NG2-Cre line it was only ~30%, ref. 50).

Depolarizing the membrane potential in rat brain slices by raising the extracellular potassium concentration ([K^+^]_o_) to 92.5 mM, which is expected to activate Cav channels without the need for TMEM16A activation, elicited a capillary constriction (Figure 5, C and D) similar to that induced by application of ET-1 (Figure 2B). Consistent with this approach, bypassing the intermediary step of TMEM16A activation, the constriction evoked by high [K^+^]_o_ was unaffected by Ani9 (Figure 5D).

### TMEM16A inhibition attenuates capillary constriction and pericyte death in ischemia.

In ischemia, low [ATP]_i_ will slow Ca^2+^ pumping out of pericytes, raising [Ca^2+^]_i_. This may activate TMEM16A and promote Cav activation. We used Ani9 to test whether blocking TMEM16A could reduce the pericyte contraction and death that ischemia evoke (6).

**Figure 6:**
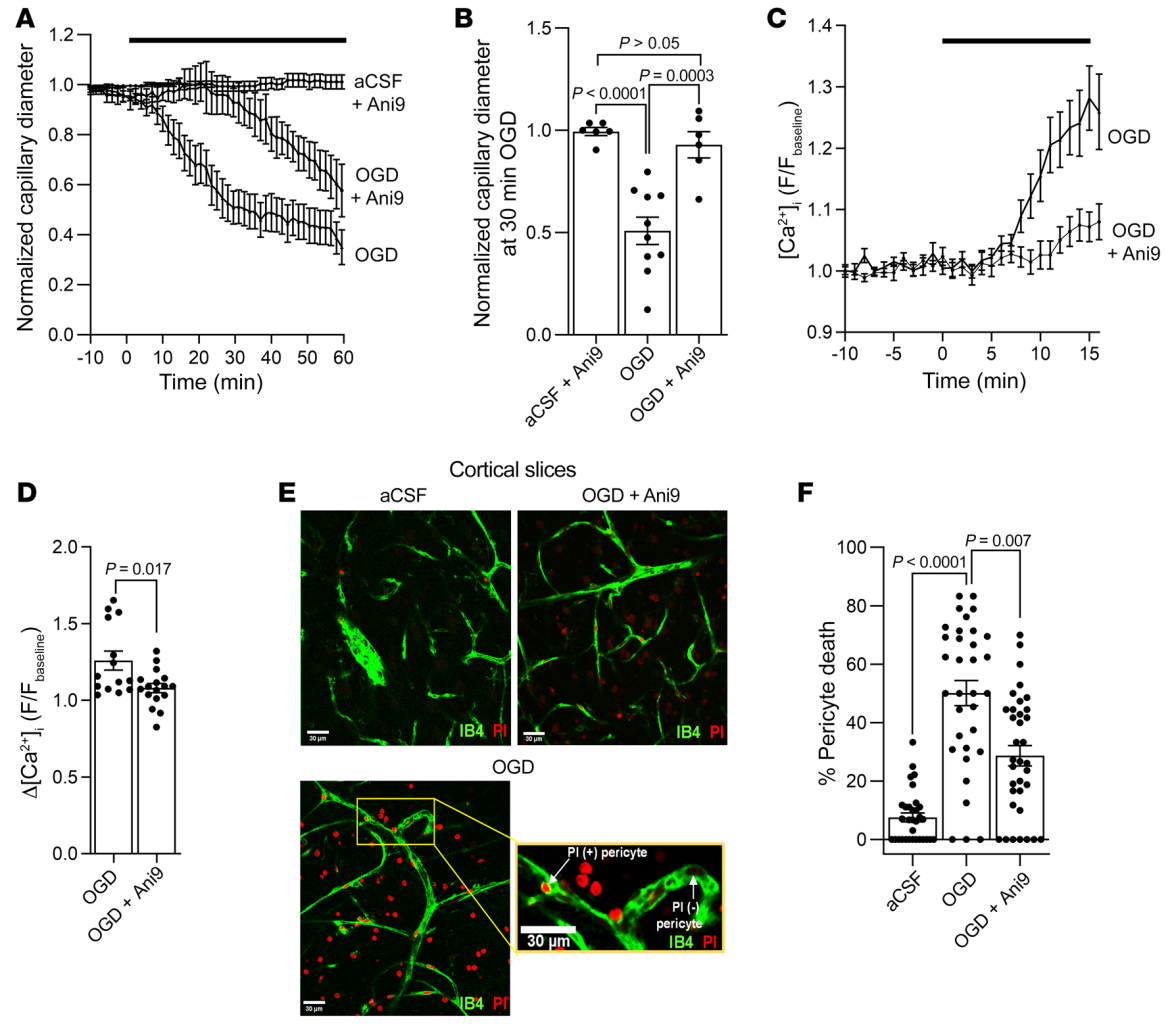
Blocking TMEM16A slows the ischemia-evoked [Ca^2+^]_i_ rise in pericytes, delays capillary constriction, and reduces pericyte death in acute cortical slices. (**A**) Mean normalized capillary diameter at pericyte somata during perfusion with aCSF (control, *n =* 6) or oxygen and glucose deprivation (OGD) solution in the presence (*n =* 6) or absence (*n =* 10) of Ani9 (2 μM) in acute rat cortical slices. (**B**) Ani9 reduces the ischemia-evoked pericyte-mediated capillary constriction at 30 minutes of OGD. Points indicate individual pericytes from 5 to 8 rats per condition (1-way ANOVA with Bonferroni’s post hoc multiple comparisons test). The OGD-evoked capillary constriction was not dependent on the sex of rats (Supplemental Figure 3B). (**C**) Time course of the mean change in normalized GCaMP5G fluorescence (F) in pericyte somata during OGD with or without Ani9 (2 μM) in NG2-Cre^ERT2^-GCaMP5G mice. (**D**) Ani9 reduces the [Ca^2+^]_i_ rise (measured from the *y* axis value of 1) at 16 minutes of perfusion with OGD. Points indicate individual pericytes (OGD, *n =* 14; OGD+Ani9, *n =* 17) from 3 mice per condition (unpaired 2-tailed Student’s *t* test with Welch’s correction). (**E**) Confocal images of rat cortical capillaries labeled with isolectin B4 (IB4) to visualize pericytes labeled by the necrosis marker propidium iodide (PI) after a 1-hour exposure to aCSF or OGD in the presence or absence of Ani9 (2 μM). The inset illustrates examples of necrotic (PI +) and healthy (PI –) pericytes. Scale bar: 30 μm. (**F**) Ani9 reduces the OGD-evoked pericyte death. The percentage of dead pericytes was quantified by dividing the number of PI-labeled pericytes by the total number of pericytes in images as shown in (**E**) (aCSF: *n =* 32; OGD: *n =* 33; OGD+Ani9: *n =* 35) (Kruskal-Wallis test with Dunn’s post hoc test). Number of animals are detailed in Supplemental Table 2.

Capillary diameters and pericyte [Ca^2+^]_i_ were measured in acute cortical slices during oxygen and glucose deprivation (OGD), in the absence or presence of Ani9 (2 μM). Ani9 strongly delayed pericyte-mediated capillary constriction (Figure 6, A and B) and reduced the OGD-evoked [Ca^2+^]_i_ rise by approximately 70% (Figure 6, C and D). Because ischemia-evoked pericyte death is partly driven by Ca^2+^ influx (5), we tested whether TMEM16A inhibition affected OGD-induced pericyte death in rat cortical slices incubated for 1 hour in artificial cerebrospinal fluid (aCSF) solution (control) or in OGD solution in the absence or presence of Ani9 (2 μM). Propidium iodide uptake was used as a marker of necrotic death of pericytes. Ani9 reduced the OGD-induced pericyte death from 50.1% to 28.7%, compared with 7.6% in normal aCSF (Figure 6, E and F).

### Genetic evidence for a role of TMEM16A in stroke.

The experiments above implicate TMEM16A in the control of capillary diameter in response to G_q_PCR stimulation and ischemia and pericyte death in ischemia. These observations suggest an involvement of TMEM16A in the capillary constriction that follows ischemic stroke. To investigate this in humans, we identified a genetic proxy for TMEM16A activity (Table 1) and applied this in a Mendelian randomization analysis (51) to explore its association with (a) the risk of developing ischemic stroke (using the MEGASTROKE genetic association study; ref. 52) and (b) recovery after ischemic stroke (using the GISCOME study; ref. 53). The genetic proxy for TMEM16A activity was a single nucleotide polymorphism (rs755016, in an intron of the *Ano1* gene that encodes TMEM16A) that associated with (a) *TMEM16A* gene expression (based on data from the GTEx database; ref. 54) and (b) raised diastolic blood pressure (based on a study of 757,601 participants; Supplemental Table 1 of ref. 55). Diastolic blood pressure was used to select the genetic proxy for TMEM16A activity because TMEM16A is known to increase systemic vascular resistance and blood pressure (56, 57). Phenome-wide association study of this proxy predominantly identified associations with psychiatric, neurologic, and circulatory traits (Supplemental Table 1), and the other traits identified may also have a circulatory component.

Our analysis showed that, while genetically proxied TMEM16A activity was not associated with increased risk of ischemic stroke (OR per allele associated with increased *TMEM16A* expression, 1.01; 95% CI, 0.99–1.03; *P =* 0.25), increased genetically proxied *TMEM16A* expression associated with worse functional outcome (score 3–6 of the modified Rankin Scale) at 60–190 days following ischemic stroke (OR, 1.13; 95% CI, 1.03–1.25; *P =* 0.01). The association of genetically proxied TMEM16A activity with outcome after ischemic stroke, but not with risk of ischemic stroke, makes effects on systemic blood pressure an unlikely mediator, as this affects ischemic stroke risk more than recovery (58). Searching the PhenoScanner database for disease outcomes or traits related to the genetic variant proxying TMEM16A activity (59) revealed no genome-wide significant associations to suggest pleiotropic effects that could bias the Mendelian randomization analysis. Thus, this analysis suggests that increased *TMEM16A* expression is associated with a worse outcome after human ischemic stroke. We therefore examined the influence of TMEM16A inhibition in an in vivo rodent stroke model.

### TMEM16A block decreases pericyte contraction in vivo and improves CBF after stroke.

**Figure 7:**
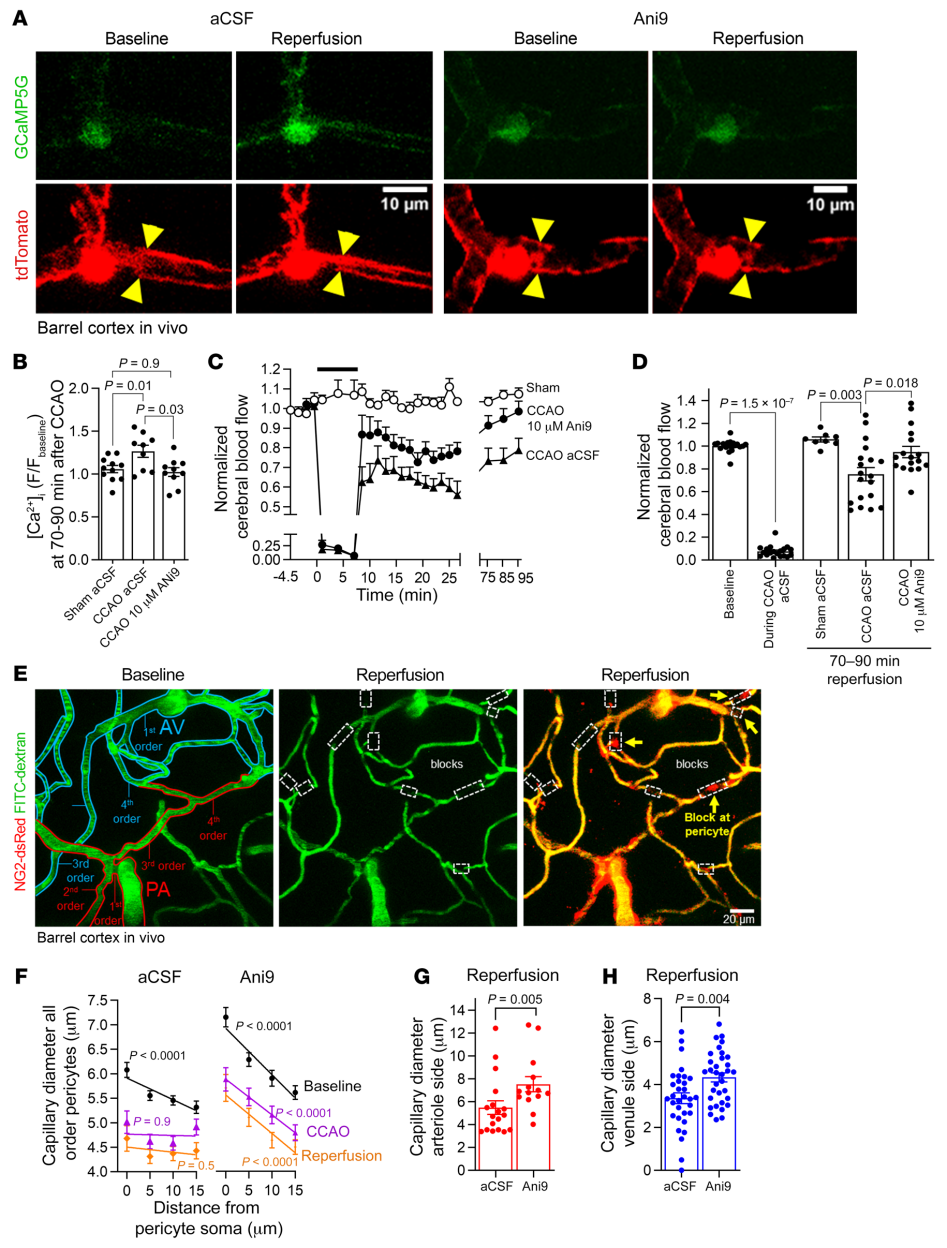
Blocking TMEM16A reduces pericyte contraction and improves CBF after CCAO. (**A**) In vivo barrel cortex pericytes in P36–P40 NG2-Cre^ERT2^-GCaMP5G mice before and after approximately 7.5 minutes of CCAO, following a 1-hour exposure to Ani9 (10 μM) or vehicle. Yellow triangles indicate sites of diameter changes. Scale bar: 10 μm. (**B**) Mean GCaMP5G fluorescence (F) in pericyte somata normalized to baseline prior to sham operation or CCAO. Data are measured at 70–90 minutes of reperfusion (sham, *n =* 11; CCAO, *n =* 9; CCAO+Ani9, *n =* 10) (1-way ANOVA, Tukey’s post hoc test). (**C**) Time course of normalized CBF from laser Doppler during sham operation or CCAO (black bar) without (aCSF, *n =* 19) or with Ani9 (10 μM, *n =* 17). (**D**) Normalized CBF at 70–90 minutes of reperfusion. Points are from individual animals (baseline CCAO, *n =* 19; sham, *n =* 8; CCAO, *n =* 19; Ani9, *n =* 17) (paired 2-tailed Wilcoxon’s test with continuity correction for “baseline” and “during CCAO aCSF” conditions; 1-way ANOVA with Dunnett’s post hoc test for other conditions). (**E**) In vivo barrel cortex of NG2-dsRed mouse with FITC-dextran in blood. Capillary branching orders are given from the PA or AV. Red and blue tracing indicate vessels on arteriole or venule side, respectively; white boxes denote occlusion sites; yellow arrows represent pericytes. Scale bar: 20 μm. (**F**) Mean baseline capillary diameter versus distance from pericyte soma for all branch orders, during CCAO and at 70–90 of minutes of reperfusion. Capillary branch orders are first to third order from arteriole (aCSF, *n =* 14; Ani9, *n =* 13); fourth to sixth order from arteriole or venule (aCSF, *n =* 4; Ani9, *n =* 7); or first to third order from venule (aCSF, *n =* 13; Ani9, *n =* 11). *P* values compare slope of the linear regression line with 0. (**G**) Mean first-order capillary diameter from PAs at 70–90 minutes of reperfusion (aCSF, *n =* 18; Ani9, 10 μM, *n =* 14). Points indicate individual capillary segments at 0–5 μm from pericyte somata (Mann-Whitney test). (**H**) As for **G**, for pericytes on first to third capillary branch order from AVs (aCSF, *n =* 32; Ani9, *n =* 33) (unpaired 2-tailed Student’s *t* test).

Bilateral common carotid artery occlusion (CCAO) was performed in mice to mimic the ischemia/reperfusion injury that occurs during severe ischemic stroke (60). Using simultaneous laser Doppler flowmetry and 2-photon imaging in vivo, we tested the effect of TMEM16A inhibition on pericyte [Ca^2+^]_i_, capillary diameter, and CBF after approximately 7.5 minutes of CCAO in the cortex (in vivo) of NG2-dsRed mice or NG2-Cre^ERT2^-GCaMP5G mice (expressing dsRed or tdTomato and GCaMP5G, under the NG2 promoter, respectively) (see Supplemental Figure 4A for the in vivo setup). Ani9 or its aCSF vehicle were applied to the barrel cortex before CCAO. CCAO resulted in an almost complete cessation of CBF (Figure 7, C and D).

Ani9 (10 μM) reduced the CCAO-evoked pericyte [Ca^2+^]_i_ rise after 70–90 minutes of reperfusion (Figure 7, A and B) and improved CBF both initially following CCAO (Figure 7C) and after 70–90 minutes of reperfusion (Figure 7, C and D). No significant change in CBF was observed in sham-operated mice (Figure 7C), suggesting that the anesthetic did not alter CBF over the duration of the experiments.

Measuring capillary diameter as a function of distance from the pericyte soma at various branch orders from the PA or ascending venule (AV) (Figure 7E) showed that the CCAO-evoked capillary constriction was largest at the pericyte somata (Figure 7F, aCSF). Before CCAO, the capillary diameter was largest at the pericyte somata, as reported in cortical slices (Figure 3E) and previously in vivo (6, 11), and capillary diameter decreased with distance from the soma, as indicated by the negative slope of the regression line differing significantly from 0 (baseline plots, Figure 7F, aCSF). CCAO and reperfusion resulted in a shallower dependence of diameter on distance, with a regression line slope that was no longer significantly different from 0 due to constriction of the capillary at the pericyte soma (Figure 7F, aCSF). Before CCAO, Ani9 treatment increased the capillary diameter at 0–5 μm from the center of pericyte somata on both the arteriole (*P =* 0.02, first to third order, Mann-Whitney test) and the venule (*P =* 0.0006, first to third order, unpaired 2-tailed Student’s *t* test) sides of the capillary bed (compare baseline plots in aCSF and Ani9 graphs, Figure 7F), suggesting the existence of some basal TMEM16A activity promoting pericyte tone in cerebral capillaries in vivo (in contrast to the brain slice data above). During reperfusion, Ani9 reduced the capillary constriction on the arteriole (Figure 7G) and the venule (Figure 7H) sides of the capillary bed, and the dependence of diameter on distance from the pericyte soma maintained a steeper negative slope that was significantly different from 0 both during and after CCAO (Figure 7F, Ani9). Thus, TMEM16A amplified pericyte-mediated capillary constriction after stroke, and Ani9 prevented this.

### TMEM16A activation enhances ischemia-evoked capillary occlusions at pericyte somata.

**Figure 8:**
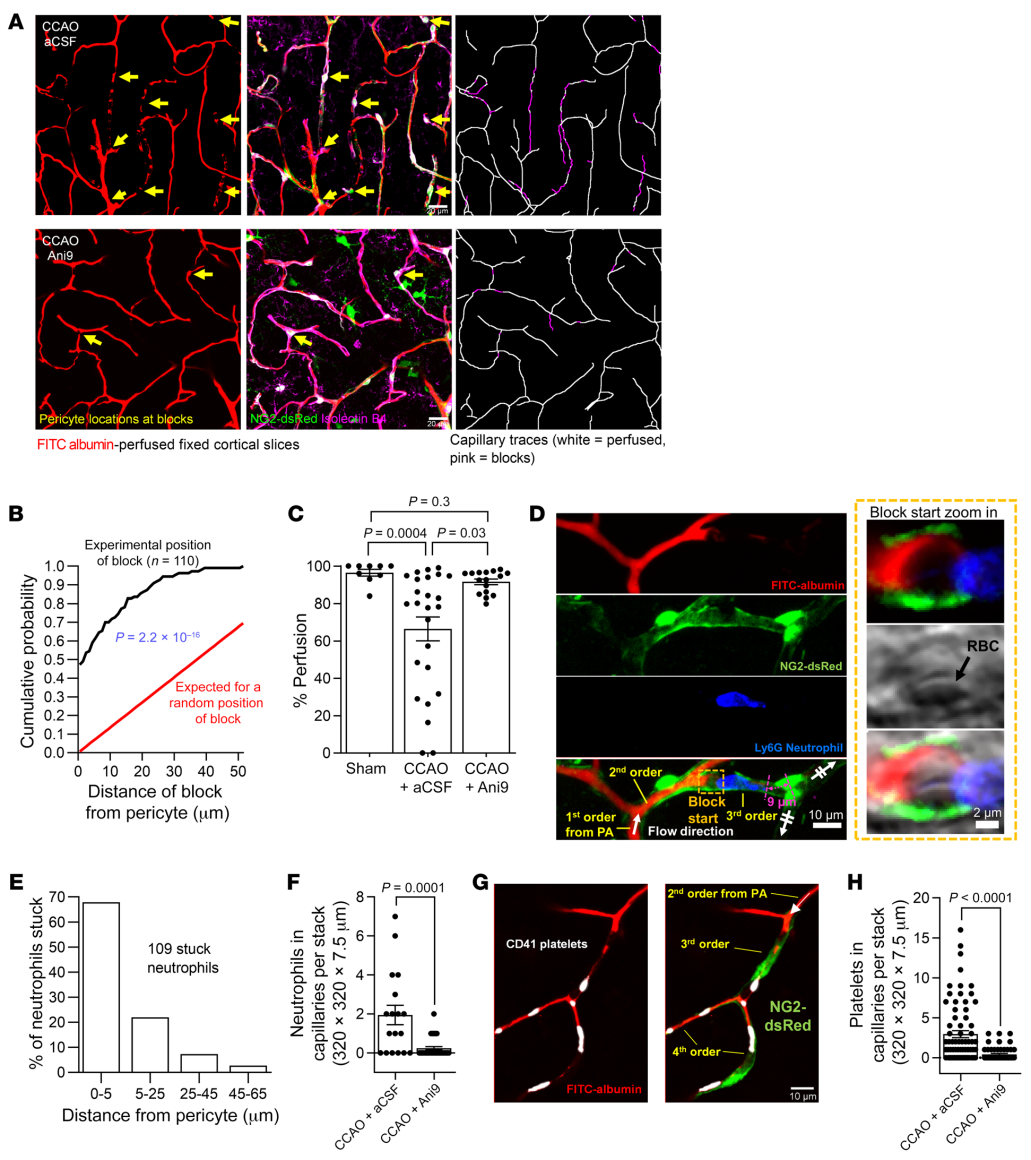
Blocking TMEM16A partially restores capillary perfusion after CCAO. (**A**) Isolectin B4–labeled cortical capillaries in fixed slices (purple); FITC-albumin in gelatin (recolored red) shows perfused vessels 1.5 hours after sham operation or CCAO without or with Ani9 (10 μM). Yellow arrows indicate pericyte somata less than 5 μm from capillary blocks. Right images show 3D tracing of FITC-albumin in perfused (white) or unperfused (magenta) capillaries. Scale bar: 20 μm. (**B**) Cumulative probability distribution of distance of 110 pericyte somata to capillary occlusions (black). The red line denotes the predicted distribution assuming pericytes are uniformly spaced along the capillary (see Supplemental Figure 5E) and blocks occur randomly (2-sample Kolmogorov-Smirnov test). (**C**) Extent of perfusion of 3D-traced capillaries. Points indicate individual confocal stacks from P30–P72 CCAO (aCSF) (*n =* 25), P31–P83 CCAO Ani9 (*n =* 16), and P39–P62 sham aCSF (*n =* 9) mice (Kruskal-Wallis test with Dunn’s post hoc test). (**D**) Ly6G-labeled neutrophil in a third order capillary in a fixed cortical slice at 1.5 hours after CCAO. The neutrophil obstructs blood flow (revealed by FITC-gelatin staining). Zoomed-in image of the right end of the block and left end of neutrophil (“Block start”) shows a possible red blood cell (RBC) to the left of the neutrophil. Scale bar: 10 μm. (**E**) Distribution of 109 neutrophils versus distance from the nearest pericyte soma. (**F**) Neutrophils in cerebral capillaries per confocal stack at 1.5 hours after CCAO in the absence (*n =* 18) or presence (*n =* 33) of Ani9 (10 μM) (Mann-Whitney test). (**G**) CD41-labeled platelets (or aggregates thereof) in third and fourth order capillary branches in a fixed cortical slice at 1.5 hours after CCAO. (**H**) Platelets (or platelet aggregates) in cerebral capillaries per confocal stack at 1.5 hours after CCAO in the absence (*n =* 71) or presence (*n =* 60) of Ani9 (10 μM) (Mann-Whitney test). The numbers of animals are specified in Supplemental Table 2.

Stalling of capillary blood flow, in part due to neutrophil block of capillaries, has been reported to decrease CBF both after ischemic stroke and in Alzheimer’s disease (16, 28, 61). We explored whether pericyte-induced narrowing of the capillary lumen during ischemia in vivo could promote transient or permanent stalling of capillary blood flow and whether this was promoted by TMEM16A activity. A FITC-albumin perfusion protocol adapted from David Kleinfeld’s lab (4, 62) was used as a tool to visualize all vessels that had been patent in vivo (see Methods). After sham operation with aCSF (*n =* 4), 7.5-minute CCAO with aCSF (*n =* 10) or 7.5-minute CCAO with 10 μM Ani9 applied locally to the cortex (*n =* 9), followed by a 1.5-hour period of reperfusion after the CCAO, we introduced FITC-albumin in gelatin into the blood, fixed the brain, and imaged sagittal sections of it. CCAO induced capillary blocks and thus evoked a perfusion deficit in the cortexes of aCSF-treated mice (Figure 8A and Supplemental Figure 5, A and B).

The cumulative probability distribution for the distance from 110 block sites in capillaries to the nearest pericyte soma, at 1.5 hours of reperfusion, is shown in Figure 8B. The mean distance was 7.2 μm for mice not treated with Ani9. There was 1 pericyte per 148 μm of vessel length (706 pericytes in 104,597 μm of vessel length traced in 10 P30–P83 mice; see Supplemental Figure 5E), so if pericytes were uniformly spaced along capillaries and the probability of an occlusion occurring was independent of position in the capillary, then the probability would be uniform from the pericyte soma to half the distance between the pericytes (74 μm, after which the occlusion would be closer to the next pericyte along the capillary). Consequently, the cumulative probability distribution would be a straight line reaching unity at 74 μm from the soma, as shown in Figure 8B. This theoretical distribution differs significantly from the experimentally observed one (*P =* 2.2 × 10^–16^, Kolmogorov-Smirnov test). Thus, the distances between occlusions and pericyte somata are significantly shorter than those expected from a random block of capillaries along their length. This is presumably because the blocks occur close to pericyte somata where capillary constriction is greatest (Figure 7F), owing to most circumferential contractile processes being located near the soma (11). TMEM16A activation, which enhances pericyte contraction after stroke (Figure 7, A and E–H), will thus increase the occurrence of capillary blocks.

3D capillary tracing was used to assess capillary perfusion, as quantified by measuring the length of vessels perfused with FITC-albumin, expressed as a percentage of the total length of isolectin B4–labeled vessels present (Figure 8A). A small perfusion deficit of 3.5% was detected in sham-operated mice, possibly reflecting naturally regressing vessels (63) or artifacts introduced by the perfusion protocol. CCAO increased this deficit to 31% (Figure 8C). Local application of Ani9 reduced the occurrence of blocks (Figure 8A and Supplemental Figure 5A) and reduced the perfusion deficit to 5% (Figure 8C).

To test whether capillary blocks contained neutrophils, we used the neutrophil-specific marker Ly6G, a glycosylphosphatidylinositol-anchored protein, to stain fixed FITC-albumin–perfused sagittal sections of mice that underwent CCAO and 1.5 hours of reperfusion after aCSF or Ani9 (10 μM) were applied to the cortical surface (Figure 8D and Supplemental Figure 5B). This revealed a neutrophil density of 1 per 1055 μm of 3D-traced vessel length and a mean neutrophil-to-pericyte distance of 6.8 μm in the cortexes of aCSF-treated mice (Supplemental Figure 5E), which is similar to the mean distance of occlusion sites from pericyte somata (see above). Some stalled neutrophils had red blood cells associated with them (Figure 8D). Plotting the distribution of stalled neutrophils as a function of distance from the nearest pericyte showed that 68% of neutrophils were within 5 μm of pericyte somata (Figure 8E). This is consistent with neutrophils becoming trapped in capillaries at contracted pericytes (refs. 16, 28, 64 and Figure 8A). Tracking the capillary branching order of neutrophils from the PA or AV in aCSF-treated mice, after 1.5 hours reperfusion, showed that 16% of stalled neutrophils were on first- and second-order capillary branches from PAs and 41% were on first- and second-order branches from AVs (Supplemental Figure 5, B–D), suggesting that the majority of stalled neutrophils are located in the middle or on the venule side of the capillary bed.

Ani9 treatment significantly lowered the number of neutrophils stalled in capillaries (Figure 8F), reducing the (presumably trapped) neutrophil density 7-fold (to 1 per 7397 μm vessel length; Supplemental Figure 5E). No neutrophils were detected in arterioles, venules, or in extravascular areas of the brain parenchyma. Labeling platelets for CD41 revealed that they also tend to occur near sites of stalled capillary blood flow (Figure 8G). Ani9 similarly greatly reduced the number of platelets stalled in capillaries (Figure 8H).

Thus, TMEM16A currents contribute to the ischemia-evoked depolarization of pericytes that facilitates capillary constriction and, hence, neutrophil and platelet stalling in the acute phase of simulated stroke.

### TMEM16A block improves CBF, reducing infarction and cerebral hypoxia after ischemic stroke in aged mice.

**Figure 9:**
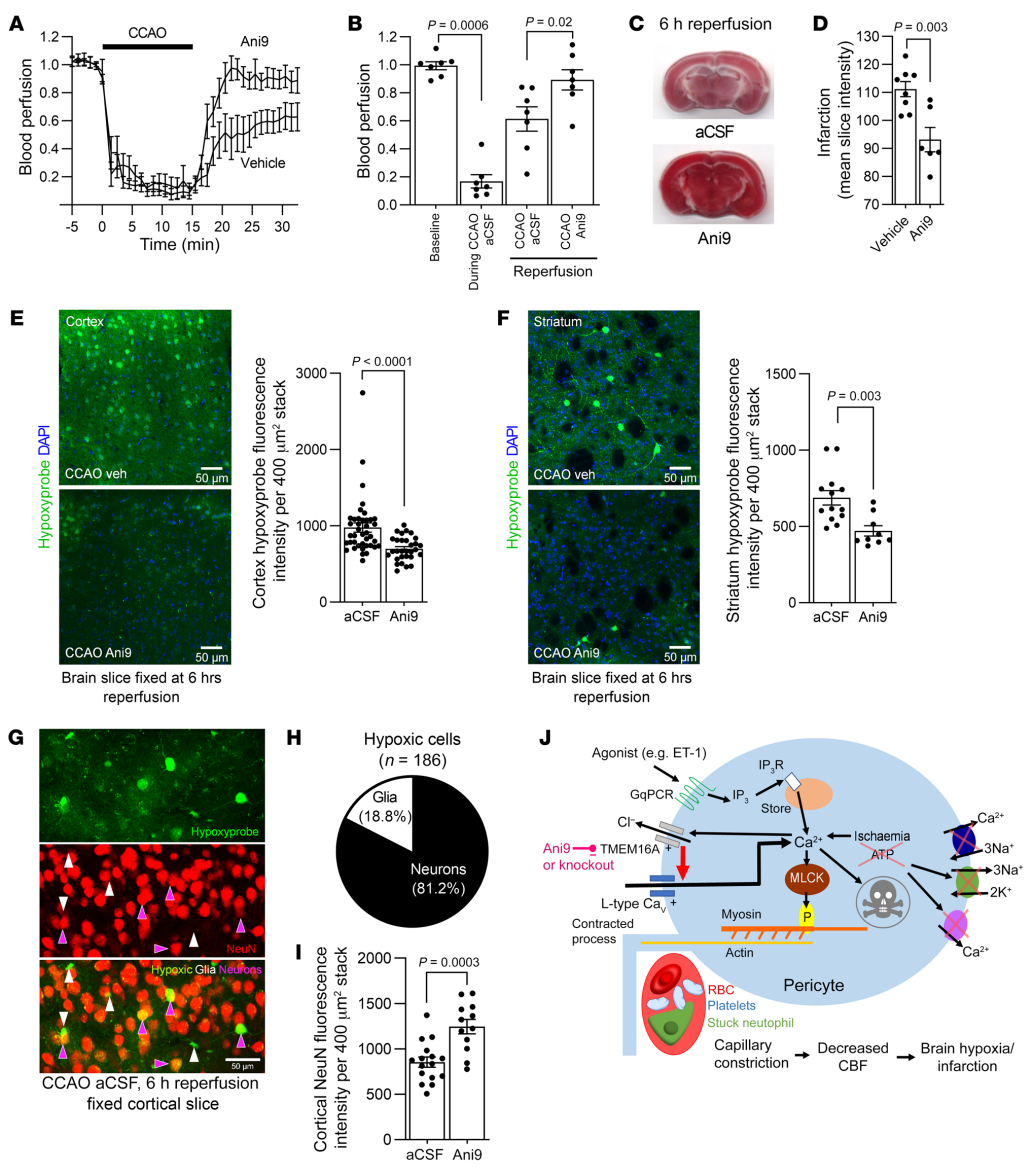
Blocking TMEM16A improves CBF and reduces neuronal hypoxia and infarct size in aged mice. (**A**) Normalized CBF from laser Doppler during CCAO (black bar) in the absence (aCSF, *n =* 7) or presence of 10 μM Ani9 (*n =* 7). (**B**) Normalized CBF during CCAO or reperfusion (average of last 5 minutes of traces in **A**) in 15 month-old mice (*n =* 7 for each, each point is 1 Doppler recording) (paired 2-tailed Wilcoxon’s test with continuity correction and Mann-Whitney test). (**C**) TTC-stained brain sections at 6 hours of reperfusion after CCAO in aged mice. (**D**) Infarction quantified from mean intensity of TTC-stained sections (Mann-Whitney test) (vehicle, *n =* 8; Ani9, *n =* 6). Confocal images of fixed cortical (**E**) and striatal (**F**) slices from aged mice that were injected with pimonidazole (Hypoxyprobe) in vivo at 70 minutes after CCAO. Mice underwent 6 hours of reperfusion. Bar graphs indicate Hypoxyprobe intensities in the cortex (**E**) and striatum (**F**) from mice treated with aCSF (cortex, *n =* 40; striatum, *n =* 13) or Ani9 (cortex, *n =* 30; striatum, *n =* 9) (Mann-Whitney test and unpaired 2-tailed Student’s *t* test). (**G**) Confocal image of layers II and III in a fixed cortical slice from a mouse undergoing 6 hours of reperfusion after CCAO. (**H**) Proportion of cells with a glial or neuronal morphology labeled with Hypoxyprobe in the cortex quantified from images, as in **H**. (**I**) Cortical NeuN fluorescence intensity at 6 hours reperfusion (aCSF, *n =* 16; Ani9, *n =* 12) (unpaired 2-tailed Student’s *t* test). (**J**) Schematic of mechanisms revealed. G_q_PCR activation triggers the IP_3_ pathway; the resulting [Ca^2+^]_i_ rise stimulates TMEM16A, cell depolarization, and Cav-mediated Ca^2+^ entry. In ischemia, low ATP slows Ca^2+^ pumping, leading to TMEM16A activation, pericyte contraction, and death. Neutrophils and platelets become trapped as pericytes contract and capillaries narrow, further lowering CBF. TMEM16A inhibition enhances capillary reflow and reduces tissue damage. Animal numbers are provided in Supplemental Table 2. Scale bar: 50 μm.

To assess whether TMEM16A inhibition confers neuroprotection in a mouse model of stroke that incorporates a major risk factor for stroke, namely aging, we performed approximately 15 minutes of bilateral CCAO in 15-month-old mice followed by 6 hours of reperfusion (see Supplemental Figure 4B for in vivo setup). Similar to the experiments carried out in younger mice, TMEM16A inhibition with Ani9 applied topically to the barrel cortex improved CBF after CCAO as compared with mice treated with aCSF alone (Figure 9, A and B). Consistent with this, TMEM16A inhibition reduced infarct size assessed using 2,3,5-triphenyltetrazolium chloride (TTC), which stains (dark red) metabolically active tissue when it is reduced by succinate dehydrogenase (Figure 9, C and D). Using pimonidazole (Hypoxyprobe Inc) to measure hypoxia after CCAO in vivo, we found that TMEM16A inhibition significantly decreased hypoxia labeling in the cortex and striatum (Figure 9, E and F). Counting the number of hypoxic neurons and glia revealed that the majority (81.2%) of hypoxic cells in the cortex were neurons (Figure 9, G and H). Furthermore, loss of cortical NeuN labeling, which is a marker of neuronal injury after ischemic challenge (65), was reduced by TMEM16A inhibition in the cortexes of mice undergoing 6 hours of reperfusion (Figure 9I).

Arterial blood pressure rises by approximately 30% during bilateral CCAO (66, 67) and then slowly recovers to baseline (67). In rodents and humans, low blood pressure has been reported to worsen neurological outcome (presumably by reducing cerebral perfusion; refs. 68, 69). The reduction of brain injury that we observed with TMEM16A inhibition (as assessed by infarct size, hypoxia, and loss of neurons (Figure 9) is unlikely to reflect a fall of systemic blood pressure because the TMEM16A inhibitor was applied topically to only the brain parenchyma, where it is expected to decrease local vascular resistance and increase local blood flow, without having a large effect on total vascular resistance and hence on blood pressure. Thus, these data suggest that TMEM16A inhibition has neuroprotective effects that extend beyond the acute phase of ischemic stroke in aged mice.

## Discussion

The key findings of this study are that (a) Cl^–^ fluxes mediated by the Ca^2+^-gated channel TMEM16A are a crucial determinant of pericyte tone; (b) TMEM16A is activated during ischemia and evokes a long-lasting pericyte-mediated capillary constriction that reduces CBF and favors neutrophil and platelet stalling; (c) genetic analysis suggests that increased TMEM16A expression is associated with poor recovery after ischemic stroke (and the genetic proxy for TMEM16A expression was also associated with other psychiatric, neurologic, and circulatory traits, consistent with its effects on pericyte function and vascular tone). These findings implicate the TMEM16A channel as a potentially new target for ischemic no-reflow, a severe clinical problem with limited available pharmacological treatments.

Historically there has been debate over the role of pericytes in controlling CBF. The Nobel Prize was awarded to Krogh in 1920 for his discovery of contractile elements (now termed pericytes) on capillaries, which act independently of SMCs on arterioles (70). Nevertheless, it has been claimed that pericytes are not contractile and do not adjust capillary diameter or CBF (71), despite the publication of movies showing pericyte-mediated constriction and dilation of capillaries in the retina (see Supplemental Video 1 in ref. 5), cerebellar cortex (see Supplemental Video 2 in ref. 6), and cerebral cortex (see Supplemental Video 3 in ref. 6). Analysis showed that this claim was based on a definition of pericytes that diverged from the historical definition (72) and included only pericytes on higher capillary branch orders, which have less circumferential processes and express less contractile proteins. Nevertheless, even these pericytes have recently been shown to exhibit some degree of contraction and participate in the regulation of CBF (8). Indeed, a systematic review by the Zlokovic group (10) assessed the papers on each side of this debate for in vitro, ex vivo (brain slice or isolated retina), and in vivo studies; they showed that 16 of 16 papers reported that pericytes were contractile in vitro, 14 of 14 papers reported that pericytes were contractile using ex vivo preparations, and 13 of 15 papers reported that pericytes were contractile in vivo. Overall 37 of 39 separate papers reported that pericytes display contractility. Thus, pericyte control of capillary diameter is now recognized to be an important determinant of CBF in health and disease (5, 6, 18) (although CBF is, of course, also controlled by adjustment of the diameter of cerebral arterioles). Underpinning this is the fact that, within the brain parenchyma (where neuronal activity can affect vessel diameter), the capillary bed may be the main component of the vascular resistance (3, 4, 73).

Contrary to our expectations, we have shown that pericyte contraction evoked by agonists that release Ca^2+^ from internal stores was largely not triggered directly by the store-released Ca^2+^. Instead, as shown in Figure 9J, store-released Ca^2+^ activates plasma membrane TMEM16A Cl^–^ channels, and the resulting depolarization caused by Cl^–^ flowing out of the cell activates Cav channels that raise [Ca^2+^]_i_ much more than occurs as a result of the store-released Ca^2+^ alone (Figure 3, B and F). This amplification of the agonist-evoked [Ca^2+^]_i_ rise results in a corresponding increase in the capillary constriction produced (Figure 4).

In Ca^2+^-free solution, ET-1 did not trigger a detectable increase in [Ca^2+^]_i_ in pericytes. This suggests that the ET-1–mediated increases in [Ca^2+^]_i_ are small and localized, but they are sufficient to activate TMEM16A. We speculate that this reflects a spatial proximity between sites of Ca^2+^ release and TMEM16A channels in pericytes (74). Deletion of TMEM16A channels from cells expressing smooth muscle myosin heavy chain has been reported to lower vascular resistance and blood pressure (56). Although that work focused mainly on arteries and arterioles, our data suggest that disruption of pericyte contraction may contribute significantly to the vascular function changes following TMEM16A KO, particularly in the brain, where the majority of the vascular resistance within the brain parenchyma is located in capillaries (3, 4).

Contraction of pericytes generating capillary constriction plays an important role in the decrease of CBF that occurs after stroke (6, 18) and in Alzheimer’s disease (11). These conditions generate an initial rise of [Ca^2+^]_i_, which is caused in stroke by a failure of Ca^2+^ pumping and possibly also a release of ET-1 and thromboxane A_2_ (20, 43) and is caused in Alzheimer’s disease by ET-1 release (11). The discovery of extra signaling steps, i.e., activation of TMEM16A channels and membrane depolarization, between this initial [Ca^2+^]_i_ rise and the final constriction of capillaries that leads to neuronal pathology suggests new opportunities for therapeutic intervention to try to maintain CBF in these conditions. Our data suggest that directly inhibiting TMEM16A (Figure 6) or manipulating the [Cl^–^]_i_ gradient to make it become hyperpolarizing rather than depolarizing in pericytes (Figure 4H) are both strategies worth pursuing in order to reduce the deleterious activation of Cav channels that leads to profound capillary constriction and an ensuing decrease of microvascular blood flow, occlusion of capillaries by neutrophils and platelets, cerebral infarction, and hypoxia (Figures 7–9). Prolonged CBF decrease eventually leads to pericyte death (6, 23). Pericyte loss leads to BBB breakdown (24–27, 75) which is reduced by inhibiting TMEM16A after stroke (76).

Therapeutic TMEM16A channel inhibitors are not yet available. Our work suggests that Ani9 could form the basis for the design of drugs suitable for use in humans and with adequate BBB permeability. The recently solved cryo-EM structure of the TMEM16A channel and determination of small-molecule binding sites may help this drug discovery effort (77–80). Elucidating the mode of action of Ani9 may aid the design of specific modulators of TMEM16A channel activity for medical therapies to address a range of neurological conditions in which pericytes restrict CBF (81, 82).

## Methods

Detailed descriptions of (a) ex vivo procedures (cortical slice preparation, imaging of capillary diameter and intracellular pericyte Ca^2+^, assessment of cell death, electrophysiology, immunohistochemistry); (b) in vivo procedures (CCAO, 2-photon imaging, cardiac perfusion for 3D capillary tracing); (c) genetic analyses; and (d) statistics are reported in the Supplemental Methods.

### Animals.

Frank Kirchhoff (University of Saarland, Homburg, Germany) provided the NG2-Cre mouse, and Walter Marcotti (University of Sheffield, Sheffield, United Kingdom) provided the floxed TMEM16A mouse, which was previously generated in-house.

### Human tissue.

Human cortical tissue was obtained from patients undergoing brain resection for glioblastoma or thymus cancer, as detailed in the Supplemental Methods. These tissues samples were provided in-house.

### Blinding.

The experimenters were blinded to conditions during the execution of the experiments and/or during analysis. The methods of analysis were established during study design, and prior to execution of the experiments, to remove possible operator bias.

### Statistics.

Statistical tests used are detailed in the Supplemental Methods. Bars show the mean ± SEM of the individual recordings. *P* values of less than 0.05 were considered significant, and all tests were 2 tailed.

### Study approval.

The use of human cortical tissue samples was undertaken with ethical approval from the National Health Service (London, United Kingdom) (REC no. 15/NW/0568 and IRAS project ID 180727), and written consent was obtained from all participants donating tissue, as detailed in the Supplemental Methods. Animal breeding, experimental procedure, and methods of killing were conducted with approval of the United Kingdom Home Office (London, UK) and in accordance with their regulations (Guidance on the Operation of Animals, Scientific Procedures Act, 1986, and subsequent amendments). Animal studies were reported in compliance with the ARRIVE guidelines, as detailed in the Supplemental Methods.

## Author contributions

NK, ZI, DA, and PT designed the research. NK, ZI, CLP, TP, PS, DG, DA, and PT performed experiments and/or analyzed data. JRR generated the floxed TMEM16A mice. HS collected and provided human cortical tissue samples. DA and PT obtained funding and supervised the research. NK, ZI, DG, DA, and PT wrote the paper. NK and ZI are co-first authors; authorship order reflects the fact that NK had a uniquely important role in driving key developments in the work.

## Supplementary Material

Supplemental data

## Figures and Tables

**Table 1:**
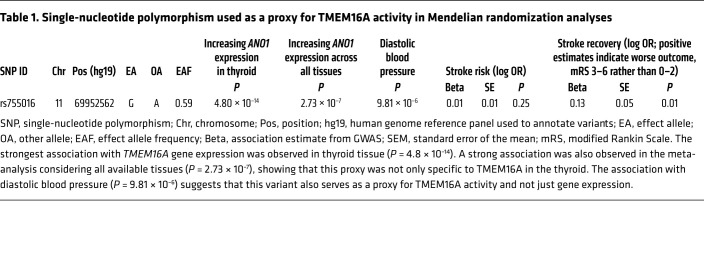
Single-nucleotide polymorphism used as a proxy for TMEM16A activity in Mendelian randomization analyses

## Version 1 03/22/2022

In-Press Preview

## Version 2 05/02/2022

Electronic publication
